# Characterization of the antagonistic secondary metabolites of *Paenibacillus polymyxa* MEZ6 against *Staphylococcus aureus*

**DOI:** 10.3389/fmicb.2025.1617807

**Published:** 2025-07-31

**Authors:** Na Zhao, Mingjiao Huang, Yang Yang, Ruxia Cai, Jian Peng, Guo Guo

**Affiliations:** ^1^Guizhou Key Laboratory of Microbio and Infectious Disease Prevention & Control, School of Basic Medical Sciences, Guizhou Medical University, Guiyang, China; ^2^Center for Tissue Engineering and Stem Cell Research, Guizhou Medical University, Guiyang, China

**Keywords:** *Paenibacillus polymyxa*, secondary metabolites, mechanism, *Staphylococcus aureus*, tryptophan-associated fraction

## Abstract

**Introduction:**

*Paenibacillus polymyxa* is an essential bio-control bacterium capable of producing numerous antagonistic compounds with potential usefulness. Methicillin-resistant *Staphylococcus aureus* (MRSA) is a significant bacterial strain that infects hospitals and communities, exhibiting considerable antibiotic resistance and posing a substantial threat to human health, thereby becoming a major bio-safety concern worldwide. The purpose of this study was to investigate the antibacterial properties and mechanisms of the secondary metabolites of *P. polymyxa* (MEZ6) against MRSA.

**Methods:**

This study used microdilution procedures and growth and bactericidal kinetics studies to investigate the effects of MEZ6 metabolites on MRSA, and reverse-phase high-performance liquid chromatography (HPLC) and mass spectrometry (LC/MC) were used to detect the secondary metabolites of MEZ6.

**Results and discussion:**

The results show that MEZ6 secondary metabolites can inhibit MRSA growth, prevent biofilm formation, reduce the expression of virulence genes (*agrA*, *spa*, and *clf-1*), disrupt cell structure, increase membrane permeability, and lead to the accumulation of ROS. Through systematic characterization, MEZ6 metabolites maybe tryptophan-associated fraction (TAF). This study establishes a systematic theoretical framework for the development and application of bacterial metabolites.

## 1 Introduction

MRSA was originally identified in 1961. It is a significant bacterium in hospitals and communities that poses a substantial threat to human health (Xu et al., 2023). Vancomycin is now regarded as an effective antibiotic for treating MRSA infections (Houkes et al., 2023). However, the long-term use of antibiotics has resulted in the evolution of MRSA resistance. Thus, the discovery of novel anti-MRSA agents carries critical importance.

*P.polymyxa* is a widely distributed, anaerobic, spore-forming, Gram-positive bacterium discovered in nature (Ash et al., 1993). This strain produces a variety of secondary metabolites, including polymyxins, enzymes, extracellular polysaccharides (*β*-glucans), nonribosomal peptides (bacilysin, inturins, rhizocticin, fengycin, and amicoumacin), ribosomal peptides (ericin S, evised sublancin), and small molecular substances with antagonistic effects (Maksimova et al., 2021; Biswas et al., 2021; Wang et al., 2020). For example, polymyxins induce membrane damage through electrostatic interactions with lipopolysaccharides (LPSs) (Khondker et al., 2019). Fusaricidins can inhibit MRSA cell wall synthesis and exhibit synergistic effects with *β*-lactam antibiotics (Rosado et al., 2025). Secondary metabolites have been widely utilized in veterinary applications, demonstrating significant antifungal efficacy against diverse pathogenic fungi. This establishes their dual importance as antimicrobial agents and environmental bioremediation tools (Vejan et al., 2016). Our analysis of *P. polymyxa* secondary metabolites provides a theoretical foundation for developing novel anti-MRSA strategies.

Here, we report the isolation of a gram-positive bacterial strain that displayed a strong lytic effect on gram-positive bacteria. The strain was identified as *P. polymyxa* strain MEZ6 based on comparisons of its 16S rRNA gene sequence and genome sequence, which revealed a high yield of secondary metabolites. This study significantly expands our understanding of the diverse characteristics of *P. polymyxa* members.

## 2 Method

### 2.1 Bacterial culture and antimicrobial activity analysis

MEZ6 was found in the soil of Meiyuan, Central China Normal University. The strain was cultivated at 30°C for 2 d in TY medium (3 g/L yeast extract, 5 g/L tryptone and 1.3 g/L CaCl_2_·6H_2_O, pH 7.0) (Zhao et al., 2022). Professor Peng Jian’s department at Guizhou Medical University provided MRSA. MRSA was cultured in TSB media (15 g/L tryptone, 3 g/L soybean papain hydrolysate, 5 g/L NaCl, 2.5 g/L glucose, and 2.5 g/L KOH, pH 7.2) at 37°C (Peng et al., 2021). The remaining strains (*Pseudomonas aeruginosa* CMCC 10104*, Staphylococcus aureus* ATCC 25923*, Escherichia coli* ATCC 25922*, Acinetobacter baumannii* ATCC 19606*, Klebsiella pneumoniae* ATCC 700603*, Cryptococcus neoformans* H99*, Candida albicans* ATCC 10231*, and Aspergillus flavus* 3357) were stored in the laboratory and cultured in SDB media (10 g/L tryptone, 5 g/L glucose, pH 7.2) at 30°C for 2 d.

The bacteriostatic zone method was used to determine the antibacterial activity of each strain. The tested strains were evenly coated on the plate, and after drying, 20 μL of the MEZ6 bacterial mixture was applied to the plate. As a negative control, the same volume of water was used. The mixture was then placed in an incubator at 37°C for 24 h for observation. Antibacterial activity is measured by the diameter of the antibacterial zone (Li et al., 2018).

For co-inoculations via different culture methods, a 2-day-old MEZ6 culture in TY medium was centrifuged at 10,000 rpm for 6 min, and the supernatant was filtered through a 0.22 μm filter. This 200 μL filtrate was inoculated into a MRSA culture (MRSA final concentration of 1 × 10^6^ CFU/mL). For cell lysate preparation, MEZ6 cells were sonicated, and the lysate was centrifuged to remove cell debris. The resulting supernatant was then added to the MRSA culture (final concentration of 1 × 10^6^ CFU/mL), and an equal volume of TSB medium was added as a control (Zhao et al., 2022).

### 2.2 Evaluation of the antibacterial activity of the isolate

The minimum inhibitory concentration (MIC) was measured using broth microdilution according to the criteria of the American Society for Clinical and Laboratory Standards Institute (CLSI) criteria (Cuenca-Estrella et al., 2010). The strains were grown to the exponential phase and washed three times with PBS (10 mM, pH 7.4). Then, 100 μL of the MRSA suspension (1 × 10^6^ CFU/mL) was inoculated into a 96-well plate, followed by the addition of 100 μL of a serially diluted MEZ6 antimicrobial material mixture, and the plate was subsequently incubated at 37°C for 24 h. The OD_600nm_ values of each well were measured via a microplate reader. The range of MEZ6 antimicrobial material concentrations evaluated was 0.2–80 mg/mL, and PBS and medium were used as negative and blank controls, respectively. The well containing the bacterial culture without drug treatment served as the 100% growth control, and the minimum drug concentration that resulted in a 50% reduction in absorbance was determined as the MIC value.

### 2.3 Scanning electron microscope observation

To study the effects of secondary metabolites on MRSA, bacterial suspensions (1 × 10^6^ CFU/mL) were treated with final concentrations of 2 × MIC or PBS for 2 h. The bacteria were rinsed twice with PBS before being fixed with 2.5% glutaraldehyde at 4°C overnight. The bacteria were then dehydrated in various concentrations of ethanol. A bacterial suspension free of MRSA was utilized as a control for comparison. Images were acquired with a Hitachi Regulus SU8100 (Tokyo, Japan).

### 2.4 Effects on cell membranes

Propidium iodide (PI, 20 μM) was used as a probe to analyze changes in the cell membrane permeability of MRSA after treatment with different concentrations of the antimicrobial substance MEZ6. The final concentration of 5 μM PI was incubated with the bacterial suspension (1 × 10^6^ CFU/mL) for 10 min, followed by treatment with MEZ6 metabolites (2.5–20 mg/mL) at 37°C for 1 h, with PBS used as a control. The fluorescence value of the bacteria was subsequently measured using a microplate reader (BioTek SYNERGY-H, Agilent, USA), with excitation and emission wavelengths set at 535 nm and 615 nm, respectively (Fadhel Abbas Albaayit et al., 2022).

### 2.5 ROS measurements

The fluorescence probe 2′, 7′-dichlorodihydrofluorescein diacetate (DCFH-DA) was used to measure the intracellular generation of ROS. The bacterial suspension (1.0 × 10^6^ CFU/mL) was mixed with DCFH-DA (final concentration of 10 mM) and incubated at 37°C for 30 min in the dark. After incubation, the suspensions were treated with MEZ6 metabolites (1/2, 1, 2, or 4 × MIC) at the indicated concentrations for 1 h at 37°C. The fluorescence intensity was evaluated using a multifunctional fluorescent enzyme marker with an excitation wavelength of 485 nm and an emission wavelength of 530 nm. N-acetylcysteine (NAC) at a concentration of 6 mM was used as a control agent to quench the production of reactive oxygen species, while PBS served as a negative control.

### 2.6 Biofilm formation

The effect of MEZ6 antimicrobial material on MRSA biofilm formation has been previously tested and described (Peng et al., 2021). An XTT test kit (Shanghai, BeatBio) was used to assess the absorbance at 490 nm via a multifunctional microplate reader after treatment with various doses of the antibacterial agent. Moreover, we used 0.5% crystal violet to measure the total biomass of biofilms at 560 nm (Vazquez-Armenta et al., 2024), with PBS serving as a negative control.

### 2.7 Alterations in nucleic acids

The experiment involved streaking MRSA strains stored at −80°C onto TSA plates and incubating them at 37°C for 1 d. MRSA strains were treated with 0, 1/2 MIC, MIC or the 4 MIC of MEZ6 secondary metabolites for 4 h, after which the supernatant was centrifuged. A UV spectrophotometer was then used to measure the absorbance at 260 nm (Hsu et al., 2005). The experiment was conducted in three parallel groups and repeated three times to ensure that the results were reliable and reproducible.

MRSA cultures were cultured in TSB overnight to assess the influence of secondary metabolites on bacterial virulence genes. The exponential phase culture was collected when the OD_600nm_ reached approximately 0.4. The cells were washed with PBS and then subjected to 1/2 × MIC for 4 h. RNA was extracted using a RNeasy Mini Kit (Qiagen, Hilden, Germany) according to the manufacturer’s instructions. cDNA synthesis and quantitative reverse transcription (RT)-PCR were performed according to the manufacturer’s instructions (Bio-Rad, CA, USA), with the primers provided in Table 1. The qPCR cycling settings were 95°C for 30 s, 40 cycles of 95°C for 5 s, and 55°C for 30 s, followed by melt curve analysis from 65 to 95°C.

**Table 1:** The qPCR primers of bacterial virulence genes.

Name	Function	Primer	Size of product (bp)
*agrA* F	Regulator of gene expression	TGATAATCCTTATGAGGTGCTT	164
*agrA* R		CACTGTGACTCGTAACGAAAA	
*spa* F	Surface protein for bacterial aggregation	GCGCAACACGATGAAGCTCAACAA	125
*spa* R		ACGTTAGCACTTTGGCTTGGATCA	
*fnb-A* F	Surface protein	ACTTGATTTTGTGTAGCCTTTTT	185
*fnb-A* R		GAAGAAGCACCAAAAGCAGTA	
*fnb-B* F	Surface protein	CGTTATTTGTAGTTGTTTGTGTT	118
*fnb-B* R		TGGAATGGGACAAGAAAAAGAA	
*clf-1*	Surface protein	CGGTTTTGGACTACTCAGCA	151
*clf-1* R		GCTACTGCCGATAAACTA	
*srrA* F	Regulator of gene expression	AGCATGTGTGGGAGGTATGA	118
*srrA* R		TGCAATCAAATATGATGTGAAGAA	

### 2.8 Isolation and purification of metabolites

The bacteria were picked from fresh plates and incubated in 50 mL of TY liquid medium at 200 rpm/min and 30°C for 2 d. The 1% seed mixture was incubated in 1 L of fermentation medium (5 g/L yeast extract, 1 g/L tryptone, 5 g/L glucose, 5 g/L beef extract and 5 g/L NaCl) for 2–3 d at 200 rpm/min and 30°C (Zhao et al., 2022). After centrifugation at 10,000 rpm for 10 min, the mixture was collected and filtered through a 0.22 μm filter.

A macroporous adsorption resin (Mitsubishi DIAION type 80*800 mm) was filled and then pretreated with ethanol and purified water, followed by fermentation broth. The samples were subsequently washed with 3 volumes of filtered water before being eluted with 50% ethanol to a light color (approximately 3 volumes) and 95% ethanol to light color (approximately 3 volumes). The 50 and 95% ethanol eluents were mixed, concentrated, and dried under decreased pressure before being separated and purified via reverse-phase separation. Then, pure chemicals were used in antimicrobial studies.

To enrich the macroporous resin, 1/5 of it was dissolved in 20 mL of 50% acetonitrile, filtered through a 0.45 μm membrane, and then injected. Mobile phase A was acetonitrile, while mobile phase B was water containing 0.1% acetic acid. The peaks with the highest purity in the crude product were collected separately and tested via HPLC. The coarse separation process described above was repeated five times. The enriched macroporous resin is coarsely separated, and then the matching flow content is merged based on the HPLC results, concentrated, and dried under reduced pressure before the target is further purified. For the elution methods, see Supplementary Table S1.

For injection, 1/3 of the crude target was dissolved in 12 mL of 40% acetonitrile aqueous solution and filtered through a 0.45 μm filter membrane. Mobile phases A and B contained acetonitrile and 0.1% acetic acid in water, respectively. The flow was collected into separate bottles and examined via HPLC. The above purification process was repeated three times, resulting in a purity of more than 95% for the target stream. Under reduced pressure, acetonitrile was removed from the target material with a purity greater than 95%. The target was then freeze-dried to provide a target with certified purity. Antibacterial tests were performed on the isolated and purified compounds. The elution methods are shown in Supplementary Table S2.

Antibacterial tests were performed on the isolated and purified compounds. Agar well diffusion was employed to examine the antibacterial activity of the extract. MEZ6 was used as a standard. One hundred microliters (1 × 10^6^ CFU/mL) of MRSA were spread evenly on agar plates. Diapers (6 mm) were created on the plates, and 100 μL of different isolated and purified compounds were loaded. The plates were incubated at 37°C for 24 h. After incubation, the apparent inhibition zone provides insight into the antibacterial potential of the isolated and purified compounds (Li et al., 2018).

### 2.9 Substance identification

The compound’s structure was determined using one-dimensional nuclear magnetic resonance (1H-NMR, 13C-NMR), two-dimensional nuclear magnetic resonance (2D-NMR), high-resolution mass spectrometry (HR-ESI-MS), Marfey’s analysis, and reference literature studies.

A checkerboard assay was used to evaluate the synergistic effects of the purified MEZ6 compounds with conventional antibacterial agents against MRSA (Yan and Hancock, 2001). Briefly, a 4 × 6 matrix was formed in a 96-well microtiter plate with twofold serial dilutions of MEZ6’s purified compounds combined with L-tryptophan (L-Trp). The fractional inhibition concentration index (FICI) was calculated as follows: FICI = MIC of compound A in combination/MIC of compound A alone +MIC of compound B in combination/MIC of compound B alone. Synergy, FICI ≤ 0.5; additive effect, 0.5 < FICI ≤ 4; antagonism, FICI > 4.

### 2.10 Statistical analysis

Every experiment was performed at least three times. Student’s *t*-test was used for statistical analysis, and *p* < 0.05 was considered a significant difference.

## 3 Results

### 3.1 Bioactivity spectrum screening

**Figure 1:**
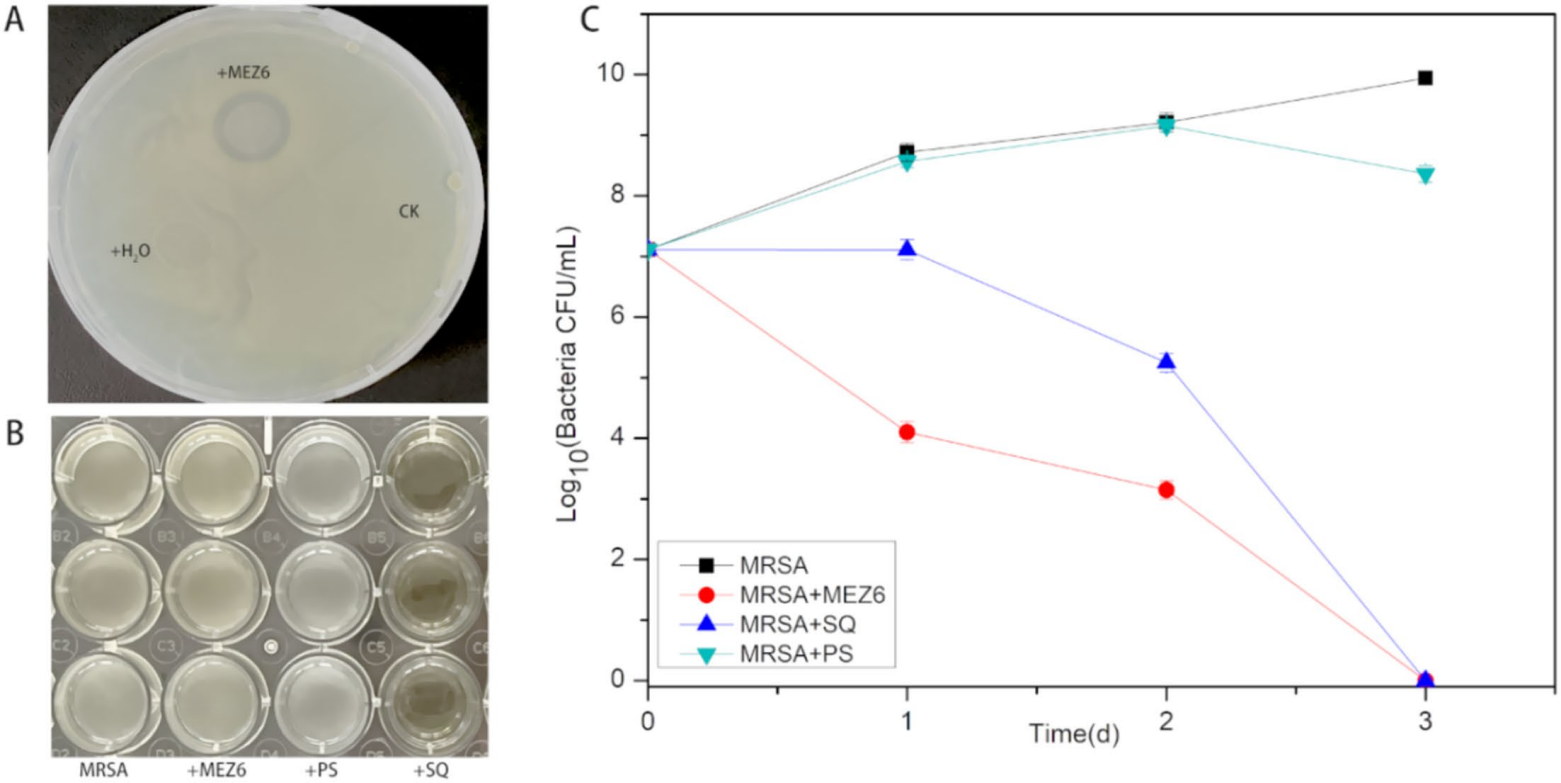
Investigation of the antibacterial mechanism of *P. polymyxa* against MRSA. **(A)** Solid plate assay. (+MEZ6: added *P. polymyxa*; +H_2_O: added sterilized water; CK: control), MEZ6 exhibited potent bactericidal activity. **(B)** Elucidating the antibacterial mechanism of MEZ6, we investigated the effects of MEZ6 on MRSA cultures (MRSA: culture containing exclusively MRSA; +MEZ6: MRSA cultures treated with MEZ6 suspension; +SQ: MEZ6 supernatant added to MRSA; +PS: MEZ6 lysate added to MRSA). The supernatant of MEZ6 demonstrated strong bactericidal efficacy. **(C)**. Time–kill curves of MEZ6, the supernatant, and the cell lysates. The bactericidal efficacy of the supernatant is comparable to that of whole MEZ6 cultures. The error bars indicate the mean ± standard deviation (*n* = 3).

MEZ6 demonstrated a substantial inhibition zone against MRSA (Supplementary Figure S1), with an inhibition rate of up to 98.98% (Table 2). In contrast, there were negligible antibacterial activity on the gram-negative bacteria or fungi examined. To further evaluate the antibacterial mechanism of MEZ6 against MRSA, antimicrobial studies were carried out using both the cell lysate and the culture supernatant. The results revealed that the active compounds preventing MRSA growth were largely found in the supernatant (Figure 1). Moreover, the minimum inhibitory concentration (MIC) of MEZ6 secondary metabolites was 5 mg/mL, and the vancomycin MIC was 2 μg/mL.

**Table 2:** Inhibition rates of the test bacteria.

Strains	Inhibition rate^a^
Bacteria	
*Staphylococcus aureus* ATCC 25923	99.6 ± 0.18%
methicillin-resistant *Staphylococcus aureus* MW2	98.98 ± 0.03%
*Pseudomonas aeruginosa* CMCC 10104	1.50 ± 2.18%
*Acinetobacter baumannii* ATCC 19606	3.41 ± 1.52%
*Escherichia coli* ATCC 25922	3.12 ± 0.75%
*Klebsiella pneumoniae* ATCC 700603	3.56 ± 2.57%
Fungi	
*Aspergillus flavus* 3357	7.18 ± 1.02%
*Cryptococcus neoformans* H99	0.58 ± 0.1%
*Candida albicans* ATCC 10231	1.06 ± 2.63%

a Values are shown as mean ± SD (*n* = 3) of three independent experiments.

### 3.2 Antibiofilm effects

**Figure 2:**
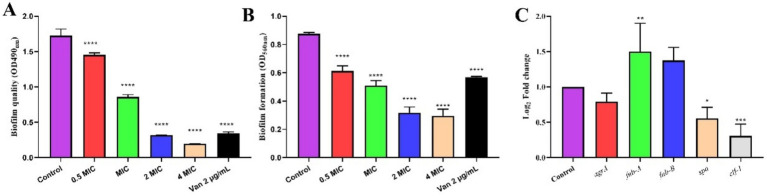
The antibiofilm effects of MEZ6 secondary metabolites (MIC = 5 mg/mL) on MRSA. **(A)**. The potential of MEZ6 secondary metabolites to inhibit MRSA biofilm attachment. **(B)**. A crystal violet assay was used to quantify the total biomass of the MRSA biofilms. **(C)**. Expression levels of virulence regulator genes (*agrA*, *fnb-A*, *fnb-B*, *spa* and *clf-1*) in MRSA. MRSA was treated with the secondary metabolite MEZ6 (1/2 MIC) for 4 h. Data represent the mean± SD (*n* = 3). *p < 0.05; **p < 0.01; ***p < 0.005, ****p < 0.001, Student’s *t*-test compared with control.

The antibiofilm potential of the MEZ6 secondary metabolites was evaluated using various methods. The XTT assay was performed to assess MRSA survival following treatment with MEZ6 secondary metabolites. At the highest concentration of 4 MIC (20 mg/mL), a significant reduction was observed (Figure 2A), and showing a dose-dependent effect on the MEZ6 secondary metabolite concentration. The results of crystal violet staining revealed that the highest concentration of MEZ6 secondary metabolites 4 MIC (20 mg/mL) significantly reduced the total biomass of MRSA biofilms (Figure 2B).

It is well known that virulence factors allow MRSA to withstand the host immune system and exacerbate infections. A thorough examination of these virulence variables will provide important insights into MRSA antibiotic resistance. The expression levels of MRSA virulence genes were detected via real-time quantitative PCR following treatment with various doses of MEZ6 secondary metabolites. Figure 2C shows that a 1/2 × MIC treatment for 4 h reduced the expression of virulence genes, including accessory gene regulator A (*agrA*), cytokine-like factor 1 (*clf-1*), and immunoglobulin G-binding protein A (*spa*).

### 3.3 Mechanism of action

**Figure 3:**
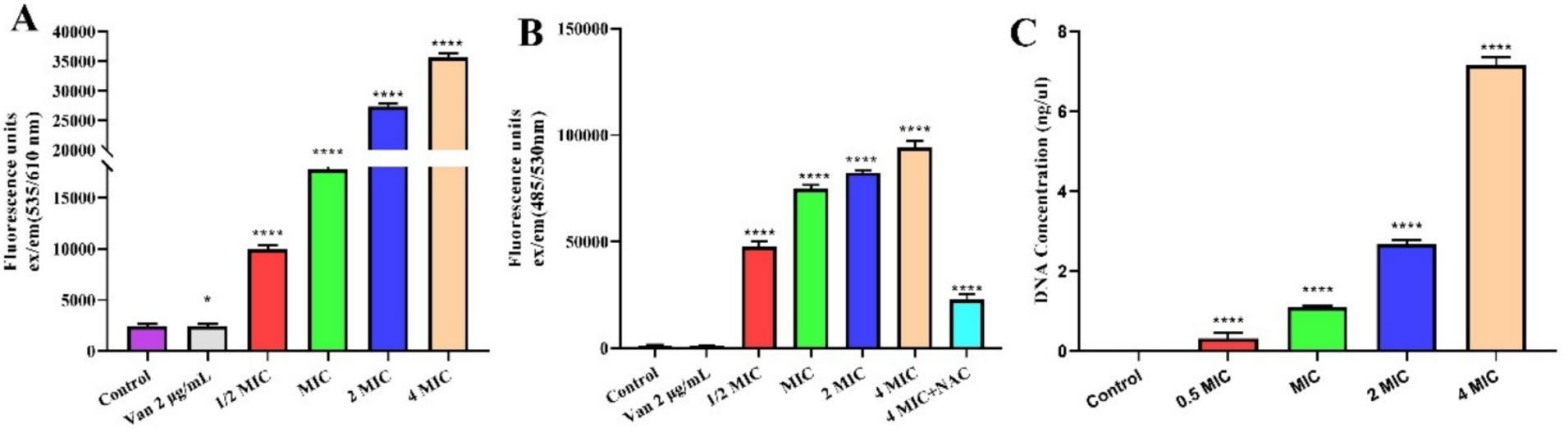
Interaction of MEZ6 secondary metabolites with the cell membrane, ROS and DNA of MRSA (MIC = 5 mg/mL). **(A)**. Fluorescence intensity of propidium iodide (PI) in MRSA treated with different concentrations of MEZ6 secondary metabolites for 1 h. **(B)**. Production of ROS in MRSA treated with different concentrations of MEZ6 secondary metabolites; exogenous supplementation with NAC (6 mM) was used as a negative control. **(C)**. DNA leakage induced by secondary metabolites of MEZ6. Data represent the mean± SD (*n* = 3). *p < 0.05; **p < 0.01; ***p < 0.005, ****p < 0.001, Student’s *t*-test compared with control.

Propidium iodide (PI) cannot permeate the intact membranes of live cells. However, necrotic cells, which have lost membrane integrity, allow PI to enter and attach to DNA. Based on this feature, PI labelling was employed in this work to distinguish between dead and live cells. Figure 3A shows that when the secondary metabolite concentration increased, so did the number of dead cells. The influx of PI indicates that MEZ6 secondary metabolites may cause damage to the MRSA cell membrane, impair membrane integrity, and increase membrane permeability. The amount of cell membrane damage was proportional to the concentration of MEZ6 secondary metabolites, indicating a concentration-dependent effect.

Reactive oxygen species (ROS) generally maintain low concentration levels within the cell, and the accumulation of higher concentrations of ROS can damage cellular structures. As shown in Figure 3B, ROS accumulation in bacteria increased significantly after treatment with MEZ6 secondary metabolites. Furthermore, the addition of the antioxidant N-acetyl-cysteine (NAC) at a concentration of 6 mM reduced the production of ROS, as shown in Figure 3B. These results suggest that the presence of MEZ6 secondary metabolites induces ROS production and may plays a key role in the antimicrobial effects exerted by MEZ6 secondary metabolites.

This study used the DNA concentration in the MRSA culture as a reference to determine the DNA content in the MRSA culture following treatment with various amounts of MEZ6 secondary metabolites. Figure 3C shows that as the quantity of secondary metabolites increased, so did the DNA content in the MRSA culture, indicating a concentration-dependent effect.

### 3.4 Scanning electron microscopy

To study the impact of MEZ6 and its metabolites on the integrity of the MRSA cell wall and cell membrane, SEM was used to examine the morphological and ultrastructural changes in MRSA cells after 4 h of treatment with MEZ6 and its secondary metabolites.

**Figure 4:**
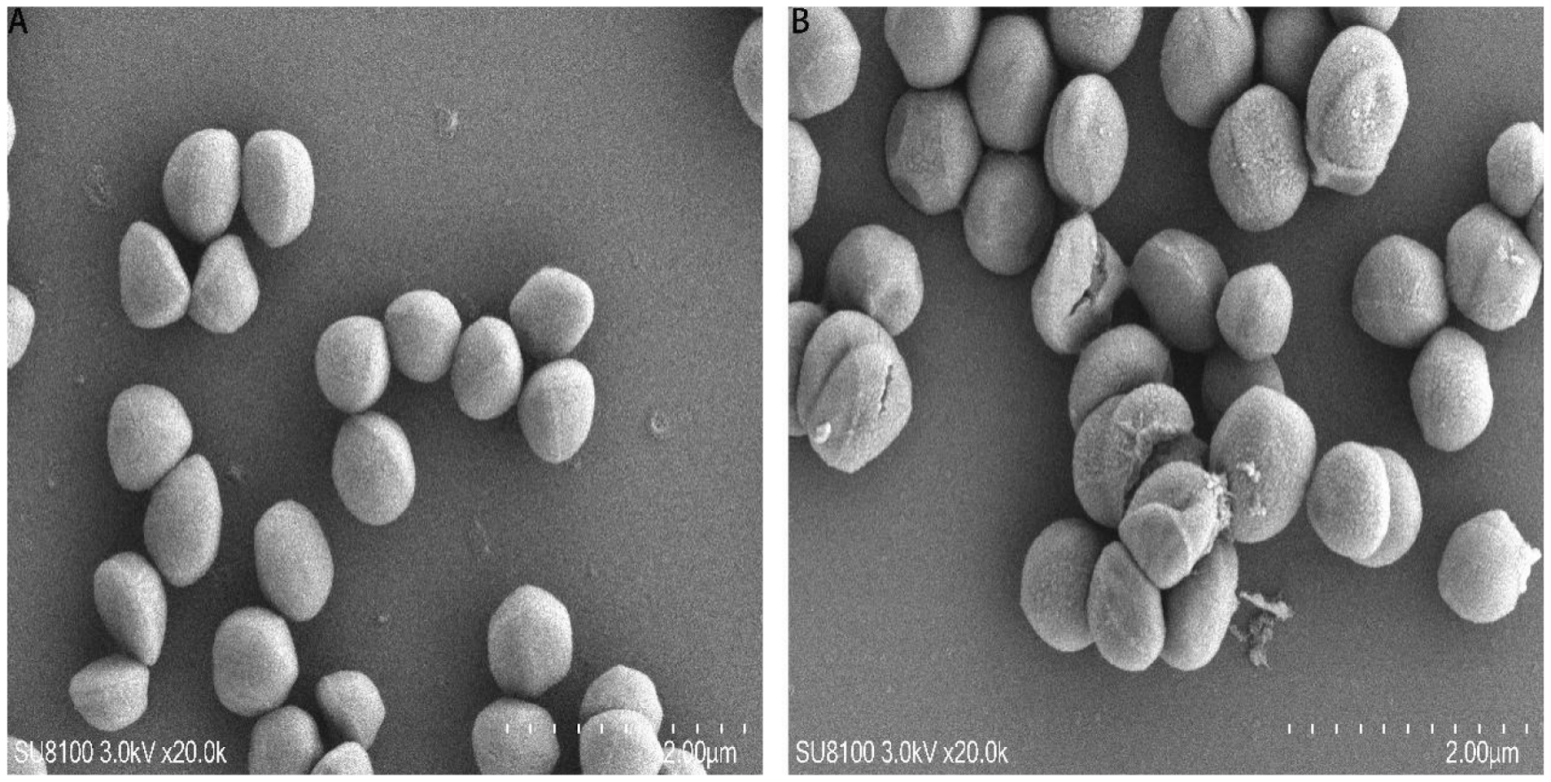
SEM analysis of MRSA. **(A)** Untreated MRSA; **(B)** MRSA treated with the MEZ6 metabolite (10 μg/mL (2 MIC)) for 2 h. MIC = 5 mg/mL.

Figure 4 shows that the MRSA cells in the control group were clear and undamaged, with a rather smooth surface (Figure 4A). In contrast, when MRSA cells were co-cultured with a 1 × MIC (5 mg/mL) of the metabolites, several cells broke, releasing their contents. The cell surface roughened, and cell aggregation occurred (Figure 4B).

### 3.5 Purification and identification of the substance

**Figure 5:**
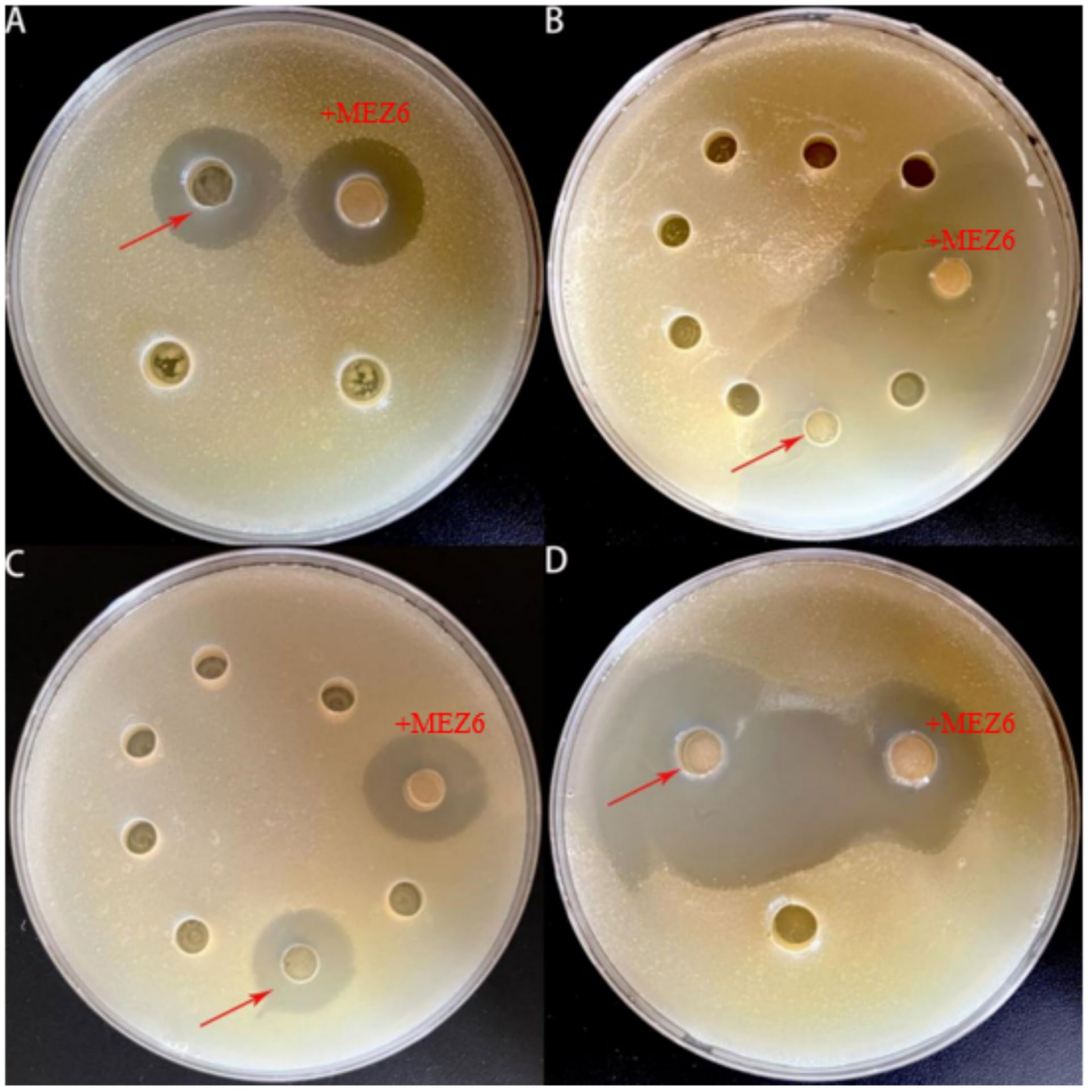
An experiment involving the isolation and purification of MEZ6 metabolites. **(A)**. Verification of 50 and 100% resin extracts. **(B)**. Continued separation and verification of 100% resin extracts. **(C)**. Continued separation and verification of the effective extracts from the previous stage. **(D)**. Verification of pure products. Each experiment was independently repeated three times.

First, the antimicrobial activity of the crude extract was tested, and the results revealed that the 100% resin extract had significant antibacterial activity (Figure 5A). The fraction exhibiting antibacterial activity was further isolated and purified, resulting in seven fractions. After their antibacterial activities were validated, it was discovered that the sixth fraction created a distinctive inhibitory zone (Figure 5B). This purified fraction was further processed, yielding six pure chemicals (Figure 5C). One of these compounds had a prominent inhibitory zone, and after further purification, a pure sample was produced (Figure 5D).

**Figure 6:**
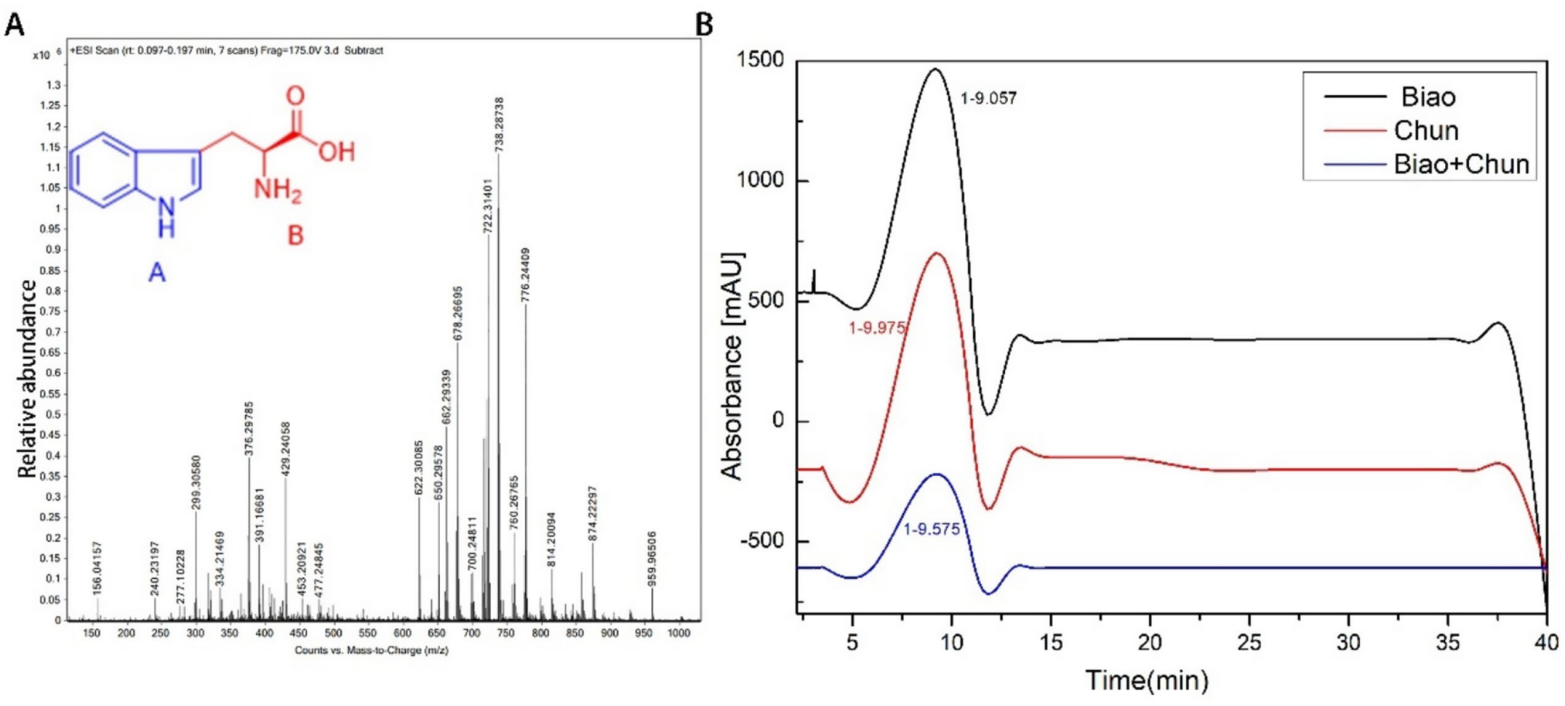
MEZ6 extracts were identified. **(A)**. Extracts were identified via mass spectrometry. **(B)**. Extracts and standards were verified via high-performance liquid chromatography (HPLC), with absorbance at 254 nm. Biao indicates tryptophan. Chun indicates TAF. Biao + Chun indicates a 1:1 (v:v) mixture of L-tryptophan and TAF. (TAF: tryptophan-associated fraction). HPLC experiment was independently repeated three times.

It reveals that TAF may serve as a key structural component in MEZ6 secondary metabolites through mass spectrometry (MS) and nuclear magnetic resonance (NMR) analysis (Figure 6A, Supplementary Figure S2–S7). We compared the pure chemical we obtained to a commercially available reference via high-performance liquid chromatography (HPLC) (Figure 6B). The results revealed that the retention periods were constant and that the peaks were solitary. We determined the minimum inhibitory concentration (MIC) of the TAF to be 3.3 mg/mL (Supplementary Figure S8). We also found that when MEZ6 pure products were combined with L-tryptophan against MRSA, the FICI value was 1.15 (0.5 < FIC ≤ 4), which indicated an additive effect (Supplementary Figure S9).

## 4 Discussion

Currently, the misuse of antibiotics is accelerating the spread of antimicrobial resistance at an alarming rate. However, microorganisms can produce structurally diverse secondary metabolites with a wide range of biological activities due to their distinct metabolic pathways and adaptation mechanisms (Maggi et al., 2024; Wallner et al., 2024), making them key sources of bioactive natural products in recent years.

*P. polymyxa* is a bacterium with numerous applications due to its capacity to enhance plant growth and produce compounds with antibacterial properties. In this study, we investigated the antibacterial efficacy of MEZ6 secretions *in vitro* against MRSA. The MIC results revealed that the inhibitory effect of MEZ6 secretions on MRSA was concentration-dependent. The secondary metabolites of MEZ6 exhibit rapid bactericidal activity. Growth curve analysis revealed that MEZ6 secondary metabolites could limit MRSA growth (Figure 1) and act via a noncontact bactericidal mechanism. These findings demonstrate that MEZ6 secondary metabolites maybe effective bactericidal agents, with effects lasting more than 2 days, providing important insights for the development of novel antibacterial agents.

To date, understanding the antibacterial processes of bacterial secondary metabolites has been difficult. It is widely accepted that they interact with membranes (Huang et al., 2025). This study demonstrates that MEZ6 secondary metabolites can significantly affect bacterial cell membrane fluidity (Figure 3).

MEZ6 secondary metabolites, like standard antibiotics, can influence bacterial activity (Figure 3A), increase intracellular reactive oxygen species (ROS) levels (Figure 3B), and cause membrane rupture (Figure 4B). Importantly, MEZ6 secondary metabolites can operate during the initial adhesion and biofilm formation stages by downregulating the expression of virulence factor genes such as *agrA*, *spa*, and *clf-1* (Figure 2C), thereby reducing the number of MRSA biofilms (Kim et al., 2012; Queck et al., 2008; Bikard et al., 2012).

Large populations of *S. aureus* in both planktonic and biofilm forms are a primary cause of chronic and recurring infections, including pneumonia, heart valve infections, osteomyelitis, and prosthetic implant infections. Our findings demonstrate that MEZ6 secondary metabolites effectively inhibit MRSA growth. Experimental data further suggest these metabolites may exhibit bacteriostatic activity across multiple MRSA growth phases. Bacterial virulence factors are crucial for the development of infections (Jenul and Horswill, 2019). *S. aureus* pathogenicity and quorum sensing (QS) are regulated by virulence genes, including *agrA*, *spa*, *fnb-A*, *fnb-B*, *clf-1*, and *srrA* (Heyer et al., 2002). The QS system in *S. aureus* uses autoinducing peptides, such as exoenzymes and exotoxins, as signals to regulate important virulence factors. In 90% of *S. aureus* infections, *the agrA* gene is responsible for causing soft tissue infections (Hauck and Ohlsen, 2006; Novick, 2003). In addition, *fnb-A* is responsible for the adhesion, colonization, and invasion of host cells (Hauck and Ohlsen, 2006; Novick, 2003), whereas *clf-1* plays an essential role in the adherence of *S. aureus* to fibrinogen and fibrin. Targeting these virulence genes could provide an interesting strategy for combating drug-resistant *S. aureus* strains (Tharmalingam et al., 2019). MEZ6 secondary metabolites downregulated the expression of *agrA*, *clf-1*, and *spa,* potentially disrupting MRSA adhesion factor function (Figure 2C).

*Bacillus polymyxa* produces polymyxin, which was isolated in 1947. Polymyxins B and E have a strong effect on Gram-negative pathogenic bacteria and are commonly used in therapeutic practice (Stansly et al., 1947). Many isolates in the genus *Paenibacillus* exhibit beneficial functions, including plant growth promotion, nitrogen fixation, biological control, and bioremediation. For example, *P. polymyxa* is frequently detected in plant rhizospheres, and through the production of NRPs (e.g., polymyxin B) or other metabolites, it can inhibit the growth of bacterial and fungal pathogens (Langendries and Goormachtig, 2021; Shaheen et al., 2011). MEZ6 exhibits antimicrobial properties similar to those of other *Paenibacillus* strains. However, there has been minimal research on the effects of the secondary metabolites of *Paenibacillus* strains against MRSA. *Bacillus* produces bacilysin to combat *S. aureus* by blocking glucosamine-6-phosphate synthase (Islam et al., 2022). JSA-9, derived from *B. polymyxa*, kills MRSA by preventing biofilm formation. Pelgipeptin D, obtained from *Bacillus elgii* B69, can kill MRSA at a 4 MIC, although the mechanism of its inhibition is unknown (Han et al., 2018). A nonpeptide component isolated from *P. polymyxa* J. exhibits substantial antibacterial action against MRSA, although the particular active ingredient has not been identified (Ding et al., 2011). This study reveals that TAF may serve as a key structural component in MEZ6 secondary metabolites.

L-tryptophan is an essential aromatic amino acid that contains an indole group and is required for humans (Yang et al., 2025). Some amino acid polyesters have been extensively investigated for use in drug delivery systems and antibacterial applications. Li et al. reported a new biodegradable wound dressing containing L-tryptophan that has bactericidal, biofilm-eliminating, and infection-preventing characteristics (Ranhotra, 2024). Trp-TOC composite materials have strong antibacterial activity against *E. coli* (NCTC-10416), *P. aeruginosa* (NCID-9016), *S. aureus* (NCTC-7447), and *C. albicans* (NCCLS 11) (Li et al., 2022). Irum Iqrar et al. discovered that bacterial extracts from *Serratia marcescens* MOSEL-w2, *Enterobacter cloacae* MOSEL-w7, and *Paenibacillus* MOSEL-w13, which are cannabis endophytic bacteria, have potent antiparasitic properties. LC–MS/MS analysis revealed that these extracts included L-tryptophan and other compounds (Iqrar et al., 2021). Tryptophan-replaced peptides of dCATH possess high antibacterial activity and cell selectivity (Feng et al., 2020). Tryptophan-rich antimicrobial peptides can kill microorganisms by targeting intracellular pathways (Mishra et al., 2018). Our study employed reverse-phase high-performance liquid chromatography (HPLC) and matrix-assisted laser desorption/ionization-time of flight (MALDI-TOF) mass spectrometry to determine the molecular weight and purity of MEZ6 secondary metabolites, indicating that MEZ6 secondary metabolites also include TAF. We also sequenced the entire genome of the bacterium MEZ6, which contains key enzymes, (e.g., MEZ6_14605, MEZ6_14610, MEZ6_14615, MEZ6_14620, MEZ6_14625, MEZ6_14630, MEZ6_14635) (Supplementary Table S3) for tryptophan biosynthesis. This also provides crucial evidence for our subsequent research on TAF maybe the key structural component of MEZ6 secondary metabolites.

The isolation of secondary metabolites from MEZ6 facilitates the understanding of bacterial metabolite functions, providing references for the development of novel antimicrobial agents. The secondary metabolites of MEZ6 may induce bacterial lysis through disruption of the cell membrane structure in MRSA. This study established a methodological system for investigating microbial secondary metabolites, with preliminary identification of anti-MRSA metabolites derived from MEZ6. However, all current experiments were conducted exclusively *in vitro* and lack cytotoxicity assessments. These limitations necessitate further *in vivo* validation to evaluate therapeutic efficacy and safety.

## Data Availability

The original contributions presented in the study are included in the article/Supplementary material, further inquiries can be directed to the corresponding author.
