# Supplementary material for: Characterization of the antagonistic secondary metabolites of Paenibacillus polymyxa MEZ6 against Staphylococcus aureus
Source: Front Microbiol. 2025 Jul 31;16:1617807. doi: 10.3389/fmicb.2025.1617807 (PMC12350257; doi:10.3389/fmicb.2025.1617807)
Supplement: Supplementary file 1 [file Data_Sheet_1.docx]

**Supplementary material**

**Table S1 Elution methods**

| Time (min) | Acetonitrile% | water with 0.1% acetic acid% |
| --- | --- | --- |
| 0 | 10 | 90 |
| 50 | 50 | 50 |
| 80 | 95 | 5 |
| 100 | 95 | 5 |

The above crude separation method was repeated five times to complete the coarse fractionation of the macroporous resin-enriched substances. Finally, the corresponding fractions were combined based on the HPLC analysis results, concentrated under reduced pressure, and dried. The target compounds were then subjected to secondary purification.

**Table S2 Elution methods**

| Time (min) | Acetonitrile% | water with 0.1% acetic acid% |
| --- | --- | --- |
| 0 | 5 | 95 |
| 70 | 35 | 65 |
| 80 | 95 | 5 |
| 100 | 95 | 5 |

Collect the fractions in separate vials and analyze them by HPLC.

Repeat the above purification method three times, then combine the target compound fractions with purity greater than 95%.

**Table S3 Tryptophan biosynthesis-related genes in MEZ6's genome**

| **Gene ID** | **Name** | **Function** |
| --- | --- | --- |
| MEZ6_14605 | *HisC* | aromatic aminotransferase or cobyric acid decarboxylase |
| MEZ6_14610 | *TrpA* | Tryptophan synthase alpha chain |
| MEZ6_14615 | *TrpB* | Tryptophan synthase beta chain |
| MEZ6_14620 | *TrpF* | Phosphoribosylanthranilate isomerase |
| MEZ6_14625 | *TrpC* | Indole-3-glycerol phosphate synthase |
| MEZ6_14630 | *TrpD* | Anthranilate phosphoribosyl transferase |
| MEZ6_14635 | *TrpE* | Anthranilate/para-aminobenzoate synthases component I |


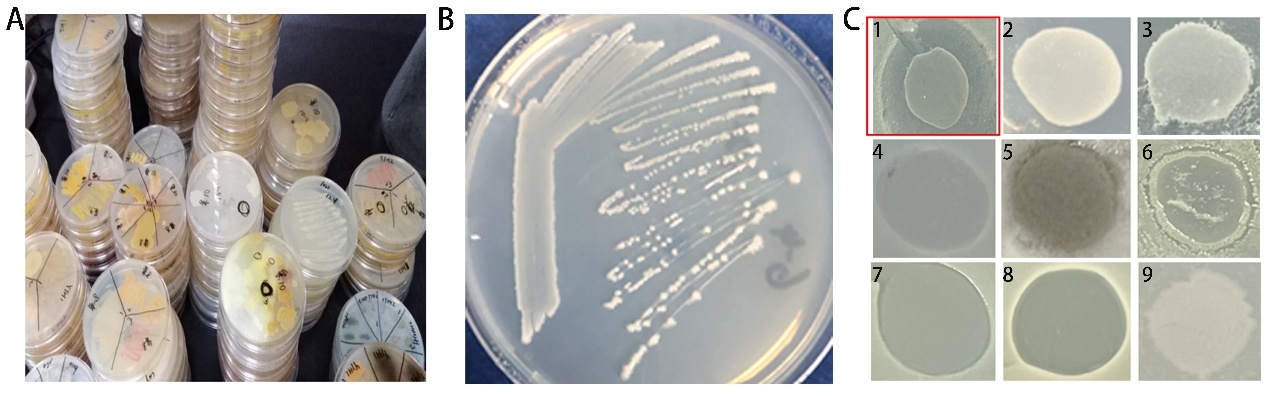


**Figure S1. Soil bacteria isolation and antimicrobial spectrum testing with MEZ6.** A. Large-scale screening of soil microorganisms was conducted, leading to the isolation of strain MEZ6. B. Solid plate colony morphology of MEZ6. C. Detection of the antimicrobial spectrum of MEZ6 (B-1: methicillin-resistant *Staphylococcus aureus* MW2; B-2: *Candida albicans* ATCC 10231; B-3: *Cryptococcus neofonmans* H99; B-4: *Pseudomonas aeruginosa* CMCC 10104; B-5: *Trichophyton mentagrophytes*; B-6: *Acinetobacter baumannii* ATCC 19606; B-7: *Escherichia coli* ATCC 25922; B-8: *Klebsiella pneumoniae* ATCC 700603; B-9: *Aspergillus flavus* 3357).


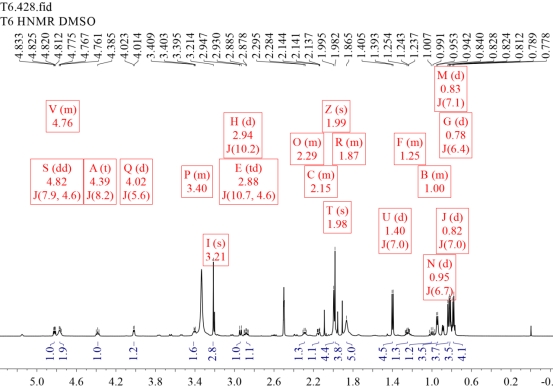

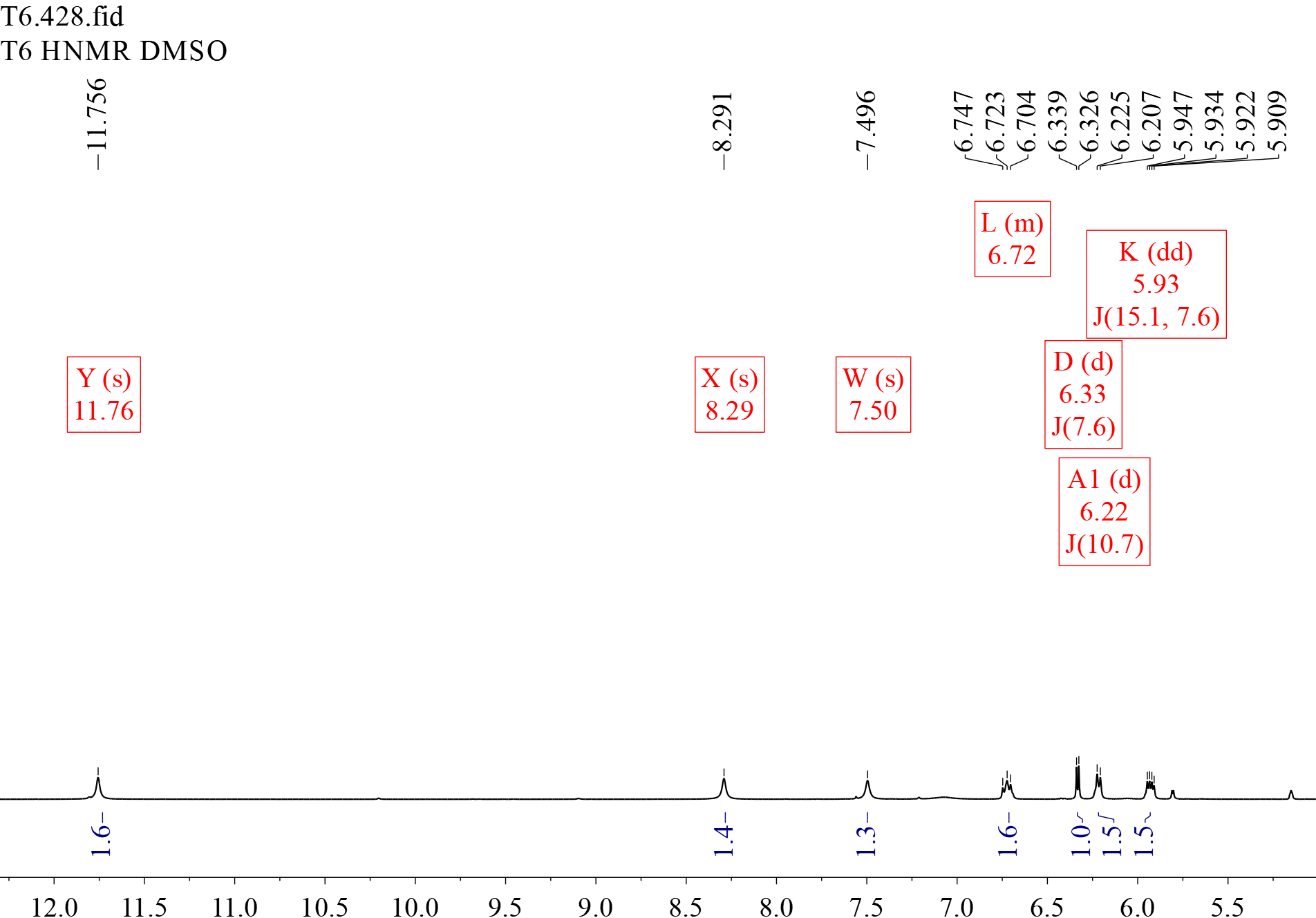


**Figure S2. ^1^H NMR (^3^H Nuclear Magnetic Resonance) of purified MEZ6.**


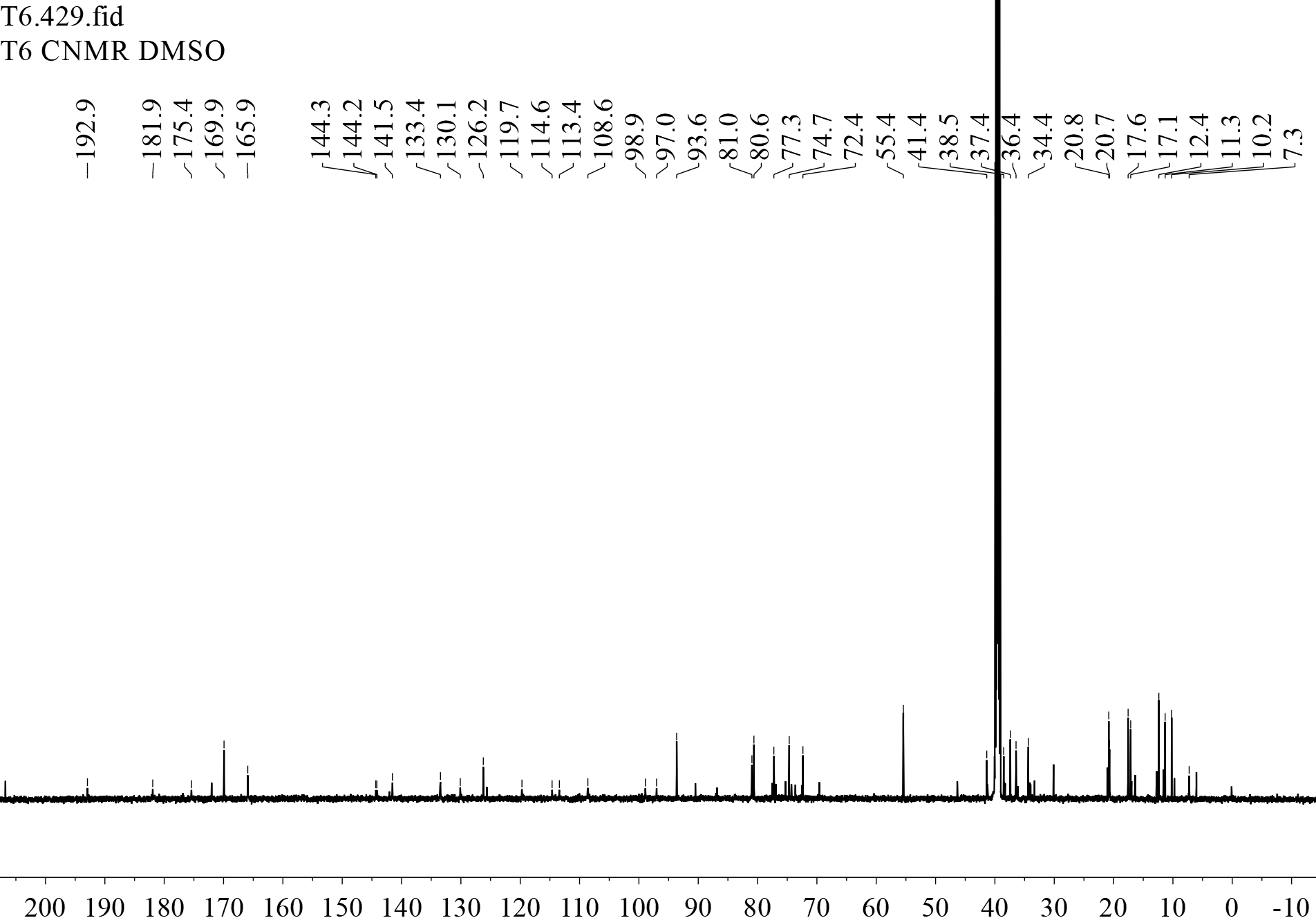


**Figure S3. ^3^C NMR (^3^C Nuclear Magnetic Resonance) of MEZ6’s purified compounds.**


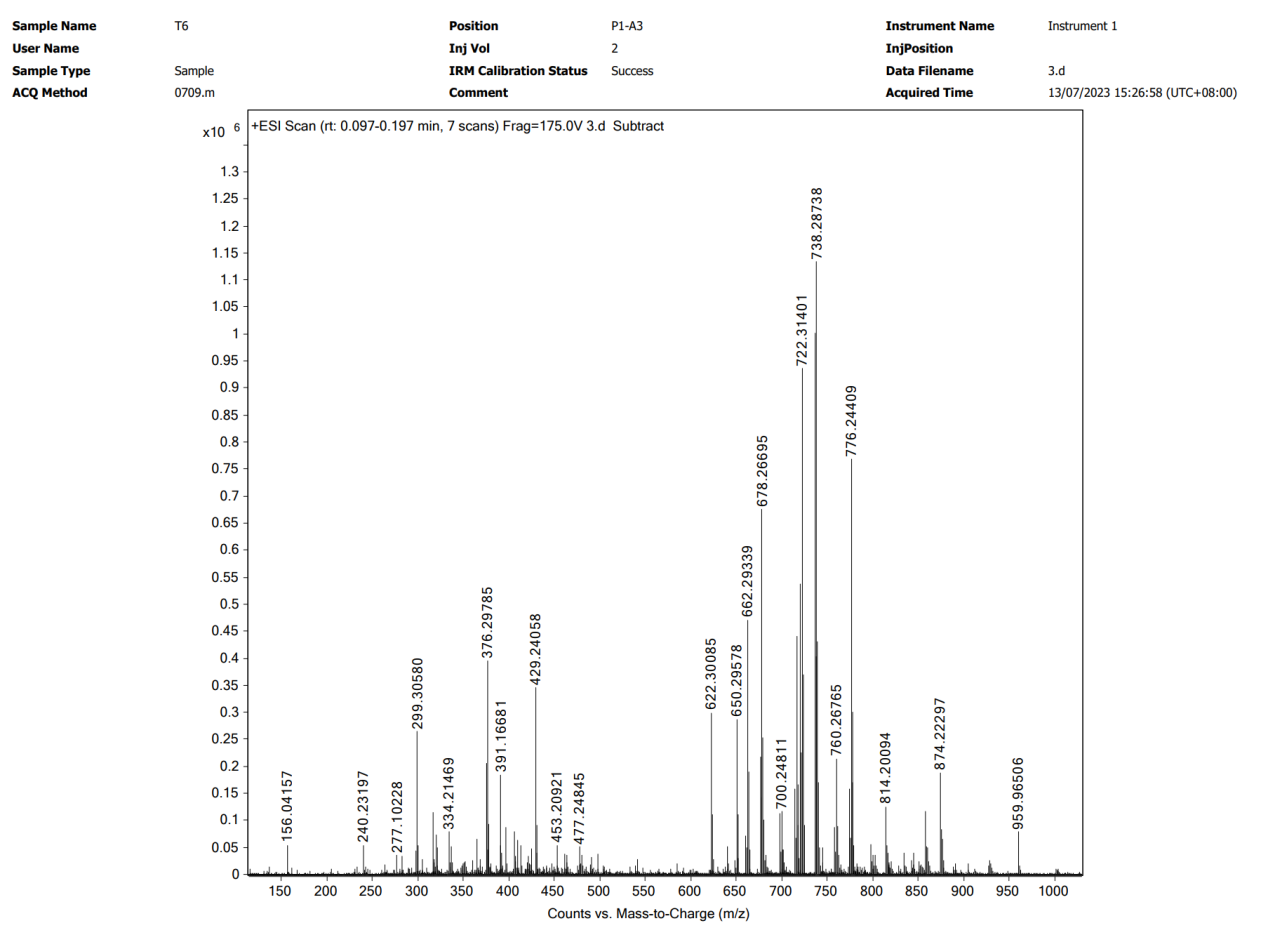


**Figure S4. Mass spectrum (positive ion mode) of MEZ6's purified compounds.**


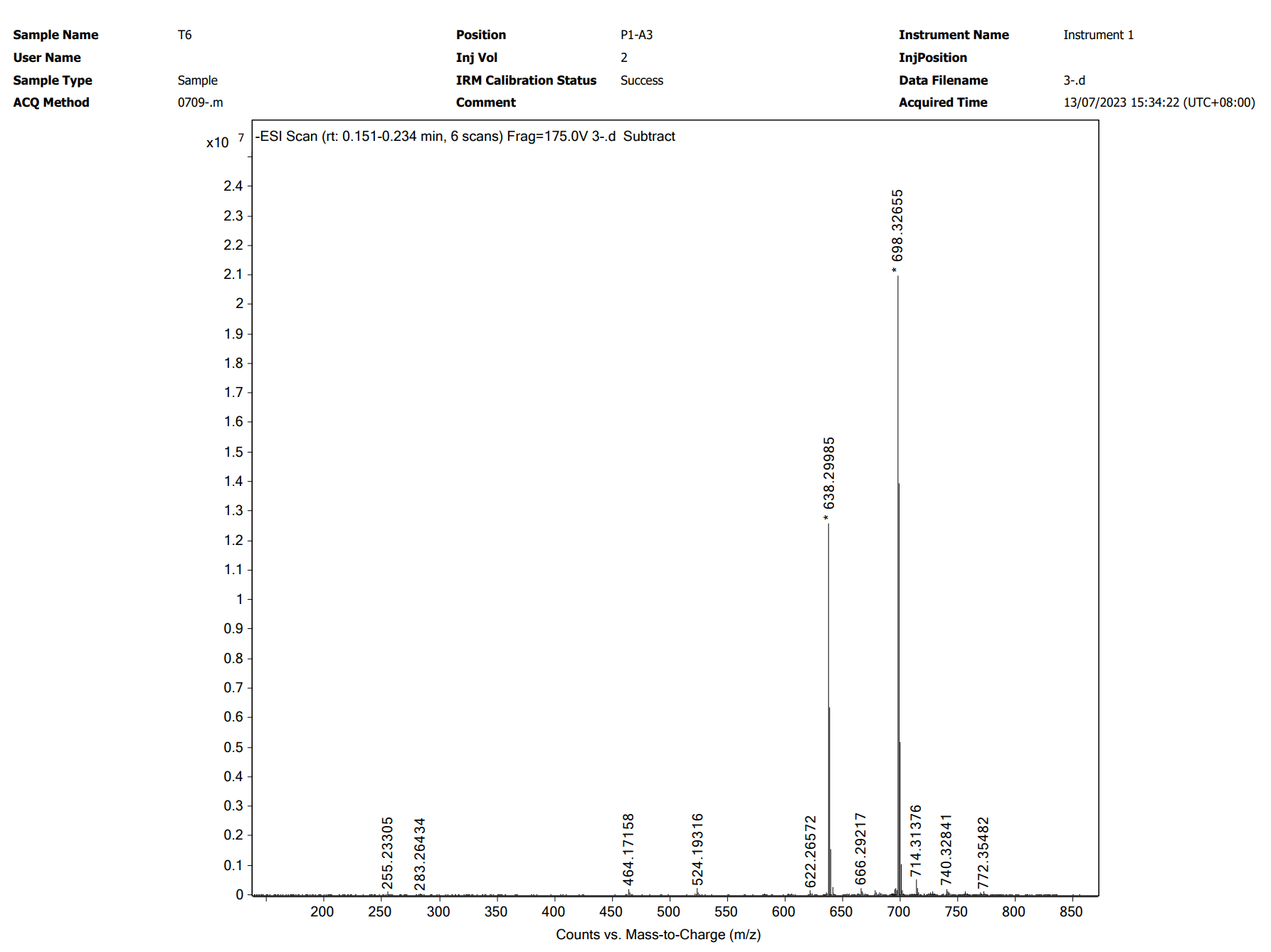


**Figure S5. Mass spectrum (negative ion mode) of MEZ6's purified compounds.**


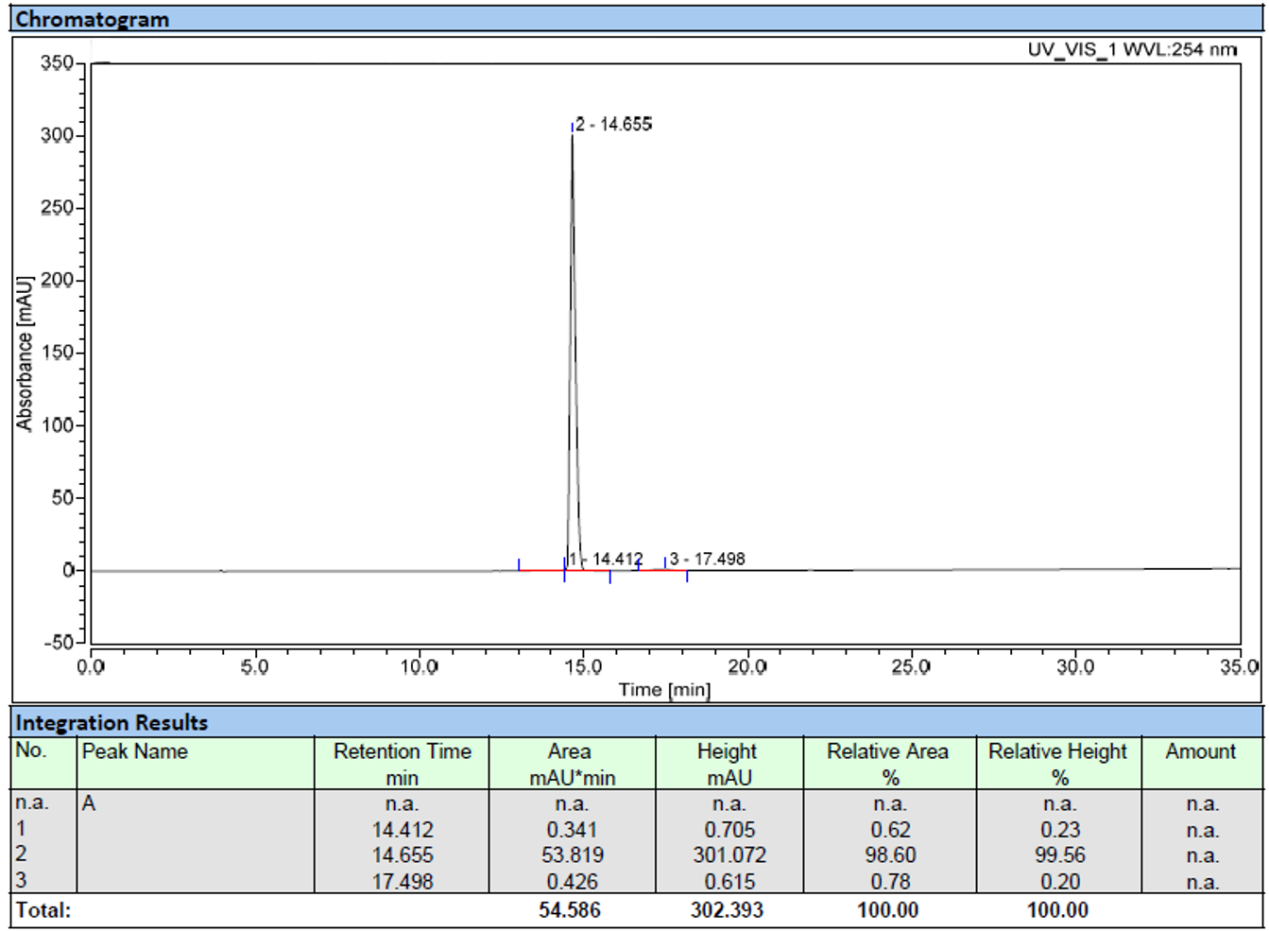


**Figure S6. HPLC (high-performance liquid chromatography) of the purified MEZ6 compounds.**

The experiment was independently repeated three times.


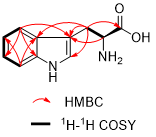


**Figure S7.** **Key HMBC and ¹H-¹H COSY correlations of purified MEZ6.**


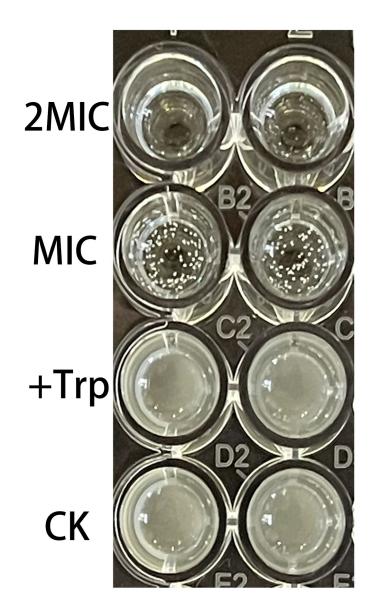


**Figure S8. Validation results of the isolated pure compounds.**

**Note:** 2MIC: adding TAF at 6.6 mg/mL to MRSA (1×10^6^ CFU/mL); MIC: adding TAF at 3.3 mg/mL to MRSA (1×10^6^ CFU/mL); +Trp: adding pure L-tryptophan (as a control); CK: as a control, containing only MRSA (1×10^6^ CFU/mL). (TAF: tryptophan-associated fraction).

The experiment was independently repeated three times.


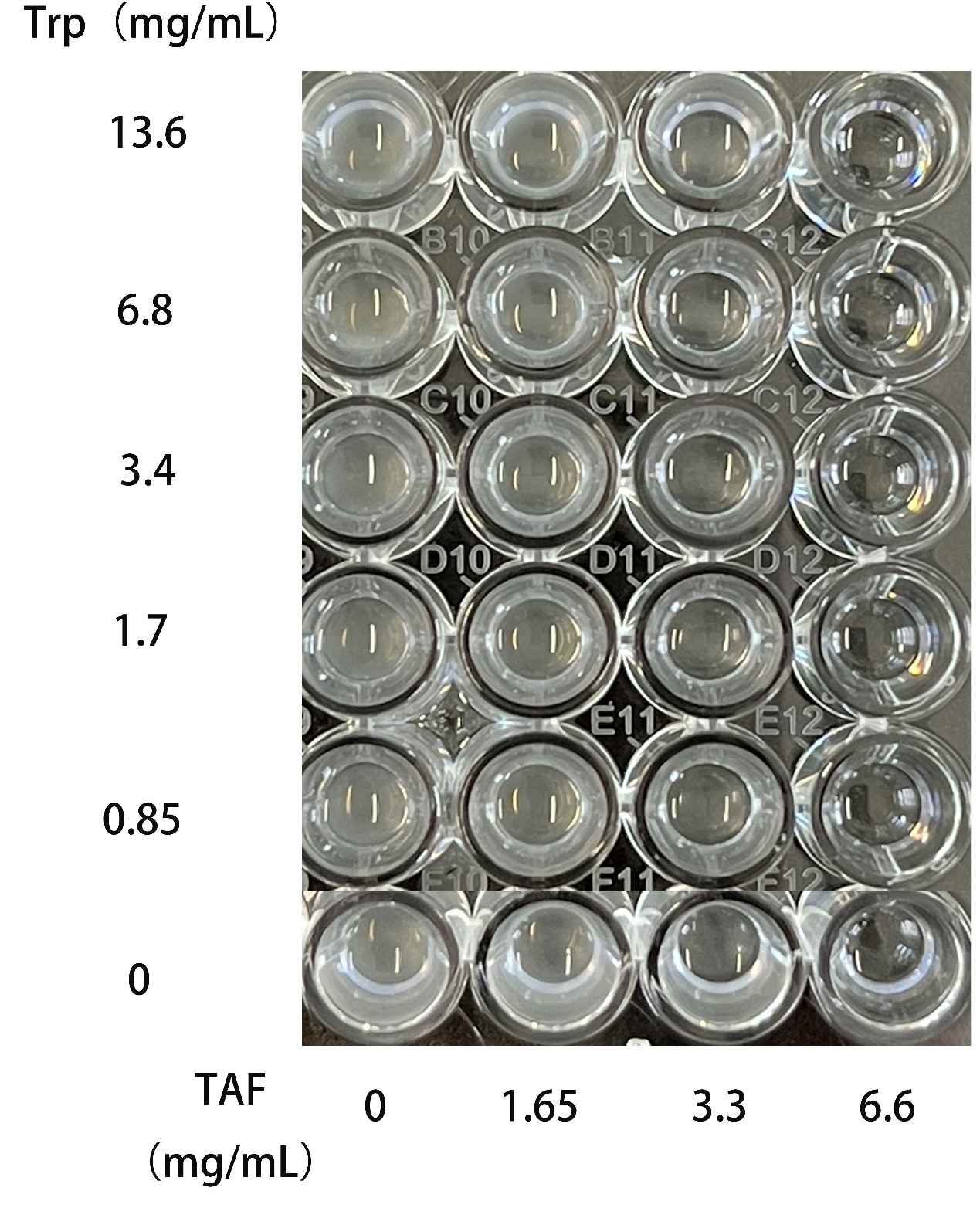


**Figure S9. Combined effects of** TAF **alone with the L-tryptophan standard.** A fractional inhibitory concentration index (FICI) ≤ 0.5 indicates synergy, 0.5 < FICI ≤ 4.0 indicates additivity, and FICI > 4.0 indicates antagonism. (TAF: tryptophan-associated fraction). The experiment was independently repeated three times.
